# Moose Space Use, Fidelity, and Selection of Calving Sites Within Forestry- and Fire-Disturbance Regimes of Northern Quebec, Canada

**DOI:** 10.3390/ani16111614

**Published:** 2026-05-26

**Authors:** Mikaela Borgeaud LeBlanc, Manuelle Landry-Cuerrier, Vincent Brodeur, Murray M. Humphries

**Affiliations:** 1 Department of Natural Resource Sciences, Macdonald Campus, McGill University, 21111 Chem. Lakeshore, Sainte-Anne-de-Bellevue, QC H9X 3V9, Canada; 2 Ministère de l’Environnement, de la Lutte Contre les changements climatiques, de la Faune et des Parcs, 951 Boul. Hamel, Chibougamau, QC G8P 2Z3, Canada

**Keywords:** Eeyou Istchee, home range, minimum convex polygons, fidelity, habitat selection, resource selection functions, manly selection ratios, generalized linear modelling, principal component analysis

## Abstract

Understanding moose calving space use in disturbed landscapes can inform forestry and wildlife management. GPS collars were deployed on 89 female moose between 2018 and 2022 in the Cree traditional territory of Eeyou Istchee in northern Quebec, Canada. We compared space use and fidelity during a 7-day period following video- and movement-estimated parturition dates to equivalent period measures in late winter and summer. We also evaluated calving site selection comparing the use and availability of terrain, land cover, road density, and fire and forestry disturbance. Female space use during the 7-day post-calving period was confined to 0.04 km^2^, comparable to 7-day space use in winter, but smaller than 7-day space use in summer. Females observed calving across multiple seasons expressed low calving site fidelity, calving a median distance of 4.00 km from previously used sites, which was lower than summer range fidelity and similar to winter range fidelity. No differences were observed in calving site space use and fidelity between a southern forestry-affected area and a northern fire-affected area or with the extent of disturbance. Female moose exhibited individual variability in calving site selection, with overall preferences for elevated areas with mixed-wood or broadleaf forests and low road densities. Females were not observed calving in areas disturbed by forestry or fires within the last year, but some females preferentially selected 10- to 15-year-old burns as calving sites. Because female moose used and remained highly localized around dispersed, annually variable calving sites, protection of moose calving habitat should move beyond the specific sites where moose are observed calving to the general habitats preferentially used by moose during this critical life history stage.

## Simple Summary

Moose calving represents a vulnerable period for female moose and their calves, and the behaviour and space use females exhibit during this time can help inform management practices aimed at conserving moose populations and their habitats. We studied the behaviour and space use of female moose during calving in the Cree traditional territory of Eeyou Istchee, northern Quebec, Canada. Using collars equipped with GPS tracking devices, we compared the extent of the areas used during calving to the extent of the areas used during other seasons, while also examining whether females returned to areas used during previous calving, winter and summer periods. We also examined what habitat types females selected during calving. Females used very small areas the week following calving, similar to areas used in winter, but smaller than areas used in summer. In all seasons, females expressed limited fidelity to previously used areas. Females preferred calving sites located in elevated areas with mixed-wood or broadleaf forests and few roads. Females were not observed calving in areas disturbed by forestry or fires within the last year, but some females calved in 10- to 15-year-old burns. These findings provide new information on the space use and habitat selection expressed by female moose during calving in northern Quebec, Canada, evidencing habitat protection priorities during this vulnerable period.

## 1. Introduction

Identifying and protecting wildlife habitat is a central component of wildlife management and conservation [1,2]. Wildlife habitat requirements include access to forage, safety from predators, and protection from weather [2,3,4]. Since forage access, predation risk, and weather exposure all vary seasonally, both in spatial distribution and relative importance, the habitats used and selected by wildlife populations often differ from one season to the next [5,6,7,8]. The seasonal specificity of habitat selection requires movement and connectivity among seasonally optimized habitats [9,10], ranging in spatial scales from localized seasonal relocations to long-range migration [11,12].

Habitats used for reproduction are critical contributors to the success of early life history stages, population dynamics, and species conservation status [13,14,15,16,17]. Animals select birth or nest sites according to features that contribute to the survival of their offspring while minimizing the costs of their parental care. Altricial young born or hatched at an early stage of development have more static and specialized birth and nesting site requirements than precocial young, which are able to move shortly after birth or hatching [18,19]. Ungulate mother-offspring associations have been differentiated into a hider strategy, often associated with forested habitats, in which mothers spend significant time away from their neonates, who remain hidden and immobile under cover, and a follower strategy, typically associated with open habitats, in which offspring move with their mothers shortly after birth [20,21,22]. Capital breeders that support reproduction with previously stored energy can select birth or nest sites prioritizing the safety of offspring, whereas income breeders need to select sites that satisfy their own foraging requirements in addition to the safety of their offspring [23,24,25]. Species occupying open habitats are more likely to nest or give birth in aggregations consisting of many other reproductive individuals [26,27], because this augments early detection, cooperative defence, and dilution effects of predation [28,29,30], whereas forest-dwelling populations are more likely to minimize predator detection by giving birth or nesting in discrete, isolated areas [31,32,33,34]. Across all these life history behavioural axes of variation, locations where individuals are born or hatch can have lasting consequences for the survival and quality of offspring, including the effectiveness and costs of the parental care they receive.

Moose (*Alces alces*) are solitary, forest-dwelling ungulates [35] that give birth to precocial calves in secluded remote areas, limiting our knowledge of moose calving behaviour and habitat use [20,36,37,38]. Moose calves are more vulnerable to predation than adults [39], from wolves (*Canis lupus*)which prey on all moose age classes throughout the year and from bears (*Ursus* spp.) that target calves during their first summer and autumn [40]. McGraw et al. [41] reported that moose remain highly localized for seven days post-parturition, using an average area of just 0.02 km^2^. Restricted or clustered space use following parturition is a movement pattern that is commonly used to identify parturition dates in moose [42,43,44,45,46] and other ungulates [47,48]. Moose calves exhibit a hider strategy during their first few days of life while their mother remains near the calving site then a follower strategy after departing the calving site [20,22,49,50]. Because female moose continue to feed throughout pregnancy and lactation [51,52], moose calving sites are speculated to reflect trade-offs between calf safety from predators and cow access to forage [37,41,42,44,53]. Predator avoidance in calving sites is often linked to cover composed of structural vegetation like trees, seedlings, shrubs, and basal growth [42]. Other moose calving site research has emphasized calf concealment offered by bogs [41] and the predator visibility and detectability offered by elevated areas [44,53,54]. McGraw, Terry and Moen [41] found that most calving sites were in bogs, followed by coniferous stands, mixed forests, deciduous forests, and regenerating habitats. Selection studies comparing moose calving site use to the availability of surrounding habitats report positive selection of gentle slopes, coniferous stands, rocky areas, and grassy meadows [55] or coniferous-dominated forests and peatland soils [56]. Among the few studies considering the multi-year fidelity of moose calving sites [57,58], Welch et al. [59] estimated an average of 3.15 km linear distance between the centres of >3-day moose localizations recorded in subsequent years. McLaren and Patterson [60] report that moose express high multi-annual site fidelity throughout the year.

Moose inhabit boreal ecosystems shaped by both forestry and wildfires [61,62,63] but the influence of these disturbances on moose calving site space use, fidelity, and selection is not well understood. Fire and forestry disturbance, combined with the regeneration trajectories and redisturbance intervals that follow, are well-documented determinants of moose abundance, distribution, and habitat selection in the boreal forest [61,62,63,64,65,66,67,68,69,70,71,72]. Wildfires and logging promote forest regeneration and create early succession habitat patches [73,74,75,76] rich in forage for moose [75,77]. Although moose generally tend to avoid recent clear-cut areas [78,79], many studies have documented preferential moose use of regenerating burns [61,64,72,80] and cuts [62,66,67,77,81,82], particularly if they occur within a landscape mosaic that includes nearby shelter provided by older and taller forest stands [67,83,84,85]. Forestry roads provide additional access to regenerating forage, including connectivity between cuts, but they also contribute to moose mortality through hunter access, altered predator movements, and vehicle collisions [67,86]. The relative effects of forestry and fire disturbance on moose calving sites remains understudied. Welch, Rodgers and McKinley [59] found that 12 female moose present in an area within a clearcut forestry expressed lower calving site fidelity (4.87 km straight-line distance between centroids of successive calving sites) than 35 females within an area with mosaic patch forestry (2.57 km). Thomas et al. [87] reported much lower probabilities of trail camera detection of females with calves in areas that experienced salvage logging in the last 10 years (0.24 ± 0.04) than in areas cut 11–25 years prior (0.83 ± 0.07) and in unsalvaged areas (0. 94 ± 0.05).

Inland portions of the Cree territory of Eeyou Istchee and southern portions of the Innu territory of Nitassinan in northern Quebec encompass spruce-feathermoss and spruce-lichen boreal forest [88]. Habitat disturbance in the area includes forestry activity in the south, and natural fire disturbance throughout the region. The collaborative Moose Habitat Quality in Eeyou Istchee Project [89,90,91] combines Cree knowledge and GPS collar analysis to evaluate the changes in moose habitat quality under an Adapted Forestry Regime implemented in 2002. Cree knowledge documented through this study emphasizes the importance of mountains, valleys, and mature and mixed forests to moose in winter, the importance of aquatic vegetation and regenerating habitats in summer, and the use of swampy and riparian zones as calving sites due to the protection they provide from predators and hunters [90]. GPS collar analyses on 38 females distributed within the Adapted Forestry Regime revealed median summer home ranges of 29.4 km^2^ and median winter home ranges of 0.97 km^2^, based on 95% minimum convex polygon calculations for 2-month periods, and showed that in both seasons females preferred mixed-wood, deciduous and coniferous forests above 7 m in height [91]. The project identified moose calving behaviour and habitat as an additional knowledge priority. Earlier documentation of Cree Knowledge, focused within the Adapted Forestry Regime, emphasized moose use of lowlands, wetlands, riparian zones or swampy regions during the calving season [92,93]. Moose fitted with GPS collars in the same region did not show selection or avoidance of swampy areas during the calving season, but did show preference for alder stands, areas near water, and low-lying locations [92].

Here we characterize moose calving site space use, fidelity, and selection in Northern Quebec based on GPS collar locations from 53 females monitored over 76 calving seasons. We quantify space use and multi-annual fidelity during a 7-day period following estimated parturition dates. We compare calving site space use and fidelity to equivalent period measures in late winter and summer to assess the distinctiveness or similarity of calving behaviour relative to other, better-studied periods of the annual cycle. We also compare the terrain, land cover, road density, fire disturbance, and forestry disturbance of calving sites relative to the availability of these habitat categories across the annual home ranges of females. Conclusions from these calving site analyses deepen our knowledge of moose calving behaviour and space use and can inform the protection of their calving habitats.

## 2. Materials and Methods

### 2.1. Study Area

**Figure 1:**
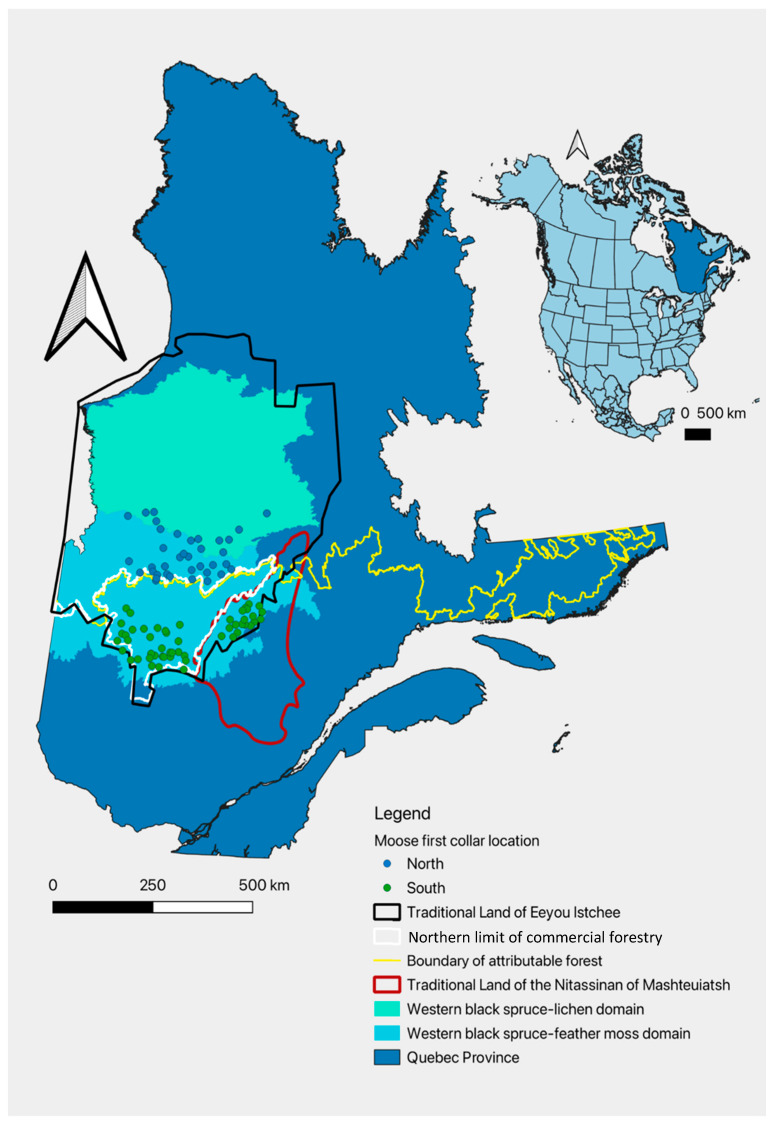
Study area located in Eeyou Istchee and Nitassinan of Mashteuiatsh, where 89 female moose were collared between 2018 and 2022, including 40 females (showing first post-collaring location) located south of the northern limit of commercial forestry and within the western black spruce-feather moss bioclimatic domain and 49 females located north of the commercial forestry including areas within the western black spruce-lichen domain. The extent of the Adapted Forestry Regime is outlined in white.

The study area, as described by Borgeaud LeBlanc, Landry-Cuerrier, Brodeur and Humphries [46], is situated within the Cree traditional territory of Eeyou Istchee and the Mashteuiatsh Innu traditional territory of Nitassinan, where the boreal forest is dominated by black spruce (*Picea mariana*) with small pockets of mixed-wood and broadleaf stands [79,94,95]. The southern portion of the study area occurs within a commercial forestry zone and the western black spruce-feather moss bioclimatic domain, whereas the northern portion of the study area is beyond the northern limit of commercial forestry and extends into the western black spruce-lichen bioclimatic domain (Figure 1) [88,96]. Forestry and forestry roads, restricted to the southern portion of the study area, and fire disturbance, distributed across the study area, have created a mosaic of habitat patches of varied stand ages and compositions that are important to plants and animals [62,75]. The severity, extent, and frequency of wildfires impacting this landscape is projected to become more intense with climate change [97,98,99]. Forestry activities occur in the Natissinan Mashteuiatsh Innu territory and the southern portion of Eeyou Istchee, below the northern limit of commercial forestry in Quebec, including within an Adapted Forestry Regime (AFR) that has been co-managed by the Cree and the Government of Quebec since 2002 [94,96,100]. As part of the AFR co-management agreement, clearcuts (250–500 ha blocks separated by 60 m residual forest strips) have been replaced by mosaic cuts (50–150 ha blocks separated by equivalent residual forest patches) and some areas are protected from forestry, including 20 m forest buffers around rivers and lakes. Sites of Special Wildlife Interests to the Cree are land-user-identified traditional use areas, often prioritizing moose hunting areas, where a smaller proportion of mature forests are harvested and a higher stand height threshold is required prior to re-harvest [100]. Within the AFR, Cree participate in forestry consultations and the spatial scale of the forest management system has been reshaped to trapline-level [100]. East of the AFR in the Innu nation of Nitassinan, commercial logging also occurs [101,102]. In 2004, First Nations of Nitassinan, the Quebec government and the Canadian government signed an agreement to negotiate a treaty that will include land management, although discussion is still ongoing [103].

### 2.2. Moose Collaring

Between 2018 and 2022, 89 female moose were collared by the Ministère de l’Environnement, de la Lutte contre les changements climatiques, de la Faunce et des Parcs (MELCCFP) [46]. Following protocols from Lamglait et al. [104], approved by an MELCCFP animal care committee, and as also described by Borgeaud LeBlanc, Landry-Cuerrier, Brodeur and Humphries [46], moose were collared after being located and darted via a helicopter either south of the commercial forestry limit or north of this extent (Figure 1). All moose were fitted with Vertex Lite GPS or GPS plus collars (Vectronic Aerospace GmbH, Carl-Scheele-Str.12, 12489 Berlin, Germany) equipped with an Iridium or Globalstar satellite communication, a very-high-frequency (VHF) beacon, a mortality sensor, a temperature sensor, an activity sensor, and a timer-controlled drop-off mechanism Lamglait et al. [104]. GPS collars were programmed to record locations at 2 h intervals for the entirety of their deployment, and locations were extracted from the onboard storage system after drop-off, thereby removing the uncertainties associated with Globalstar fix success, with no subsequent data screening or outlier removal in preprocessing. Eight of the 89 females were collared with animal-borne video and environmental data collection systems (AVEDs), which included all the elements of the GPS collars but also a camera programmed to record eight 20 s videos at 2 h intervals during approximately 16 daylight hours (off between 1:00 and 8:45 UTC, corresponding to 20:00 and 3:45 EST). Collars were programmed to drop-off between 18 and 22 months after deployment; however, the actual amount of time collars recorded moose locations and videos varied between 3 and 30 months. As a result, locations extended across multiple calving seasons for some, but not all individuals. Location data from the GPS collars were provided to McGill researchers via a data-sharing agreement with the MELCCFP.

### 2.3. Parturition Dates

Borgeaud LeBlanc, Landry-Cuerrier, Brodeur and Humphries [46] used video collar observations to assess the accuracy and precision of six movement-based methods used to estimate parturition dates. Here, we used the three movement-based methods that Borgeaud LeBlanc, Landry-Cuerrier, Brodeur and Humphries [46] identified to be the most accurate and precise to infer parturition dates from GPS collar locations. Movement methods used included the movement rate by time (MRT), which detects periods of reduced movement speed between successive locations [47], individual-based movement (IBM), which uses an automated algorithm to identify a decrease in movement distance [48], and the temporal controller, which employs the QGIS temporal controller to animate movement in space and time to determine when movement becomes localized [43]. In this study, we restricted our sample to females with more than 700 locations documented during the May and June calving period (given 2 h fix rate should generate 12 locations per day and 732 locations during the 61-day period from 1 May to 30 June a ≥700 location threshold excluded females with >5% missed fixes). We also restricted our sample to females for which the three movement-based methods yielded parturition date estimates that differed by less than four days. The parturition date used in our analysis was the average of the dates generated by the three methods. These two restrictions reduced our sample size from the original 89 females that were collared to 53 females over 76 calving seasons. To avoid pseudoreplication, analyses of statistical significance were restricted to one randomly selected calving event per female, resulting in the inclusion of 53 calving seasons for 53 females for space use and selection analyses. Additional information on movement-based estimation and AVED confirmation of moose parturition dates, calving rates, and pre- and post-parturition movement rates can be found in the work of Borgeaud LeBlanc, Landry-Cuerrier, Brodeur and Humphries [46].

### 2.4. Space Use

We used 95% minimum convex polygons (MCPs) with the QGIS (version 3.30) animove plugin [105] to quantify and compare space use. Annual home range size was estimated using GPS collar locations from 1 March to 28 February of the following year, or, if location records stopped prior to this endpoint, by using the last recorded location and retrieving data from the preceding 12 months. Calving space use was estimated using collar locations during a 7-day calving period, which included the parturition date and the next 6 days. This 7-day window was used to define the calving period based on the duration of the post-partum localization observed by Borgeaud LeBlanc, Landry-Cuerrier, Brodeur and Humphries [46] and McGraw, Terry and Moen [41]. We compare calving space use to other periods of the year, using an equivalent 7-day time window and a similar number of locations, to assess the distinctiveness or similarity of calving behaviour relative to other, better-studied periods of the annual cycle. We estimated late winter space use for the 7-day period from 21 March to 27 March. This date range was selected to maximize the number of females with locations, related to the variable timing of winter collar deployments, while still coinciding with late-winter conditions. We also estimated summer space use for the 7-day period from 1 August to 7 August. We also quantify annual space use as a longer-term, annual home-range point of comparison and because annual home range areas were used in two subsequent analyses, including the assessment of habitat availability in calving site selection analyses and the extent of forestry and fire disturbance in home range areas surrounding calving sites. We compare seasonal space use using medians as well as means because some distributions include outliers or tendencies towards non-normal distributions. We tested for study area and seasonal differences in space use using, respectively, an independent *t*-test and a one-way ANOVA followed by post hoc Tukey’s HSD. In a supplementary analysis, we compare 95% MCP estimates of calving, summer, and winter space use to 50% and 95% kernel density estimations (Supplementary Material S1).

### 2.5. Fidelity

We assessed calving site fidelity for 19 females (5 south and 14 north) that were collar- located throughout May and June of two subsequent years (with ≥700 locations) and had a calving event detected in both calving seasons by three movement-based methods (with parturition date estimates varying by ≤4 days across the three methods). We measured calving site fidelity by computing the Euclidean distance between subsequent year calving sites, with calving site location defined as the centroid of the female’s 7-day calving home range. We focused on the centroid across the 7-day calving period rather than the estimated calving location because (i) we had lower confidence in our ability to determine the precise day, time, and location of the parturition event than we did in our ability to quantify space use over a multi-day period including and following the estimated parturition date, and (ii) space use was highly restricted after parturition. To assess the distinctiveness or similarity of calving site fidelity to other, better-studied periods of the annual cycle, we calculated winter and summer home range fidelity according to Euclidean distance between the centroids of 21 March to 27 March and 1 August to 7 August space use, respectively. We also measured between-season, within-year relocation distances by calculating the Euclidian distance between the centroids of subsequent season 7-day home ranges. We compare seasonal fidelities and between-season distances using medians as well as means because some distributions include outliers or tendencies towards non-normal distributions. We tested for study area and seasonal differences in fidelity and between-season relocation distances using, respectively, an independent *t*-test and a one-way ANOVA followed by post hoc Tukey’s HSD tests.

### 2.6. Calving Site Space Use Size and Fidelity in Relation to Forestry and Fire Disturbance

We evaluated how forestry and fire disturbance related to calving site space use and fidelity, focusing on forestry in the southern study area and fire in the northern study area. Disturbance data were derived from a 30 m resolution, Landsat-based forest change product developed by Hermosilla et al. [106] and updated by Pelletier et al. [107], which identifies annual forestry and fire events across Canada’s forested landscapes from 1985 to 2022. We extracted disturbance data from the 15 years preceding each calving season (covering maps from 2003 to 2022). We defined the calving site according to parturition date location and calculated the proportion of <15-year-old disturbed area within a 6 km radius, which approximates the median annual home range size of 116 km^2^. We then used linear regression to examine the relationship between this forestry disturbance metric in the south and fire-disturbance metric in the north and either calving home range size or site fidelity.

### 2.7. Calving Site Selection Analyses

Our calving site selection analysis focused on forest stand type, permanent waterbodies, topographical landforms, and road density. Mapping of forest stand type and waterbodies was based on Hermosilla et al. [108] 30-m resolution Landsat-derived annual land cover maps from 1984 to 2022 for forested ecosystems across Canada, generated using Landsat composites, disturbance information, and environmental variables, with post-processing via a Hidden Markov Model to ensure consistent land cover transitions over time. We focused our calving site selection analysis on five vegetation cover categories (coniferous, broadleaf, mixed-wood, shrubs, other) and three permanent waterbody categories (onshore or on land within 80 m of shoreline, nearshore or in water within 25 m of shoreline, and offshore or in water more than 25 m away from shoreline). We focused our calving site selection analysis on six topography categories (peak ridge, upper slope, upper slope flat, lower slope, lower slope flat, valley) sourced from Theobald et al. [109]. Mapping of road density was based on Gouvernement du Québec [110] polyline road network map, available as a routard road layer within Forêt ouverte Quebec database. We focused our calving site selection analysis on three road density categories (low, medium, high) calculated using polyline density over a 1 km radius and categorized using Jenks natural breaks. Habitat variables were clipped to the study area extent and aggregated from original categories and sources (Table S2.1) with ArcGIS Pro 3.0 [111].

We assessed selection of calving site characteristics relative to the annual home range, employing a used versus available study design focused on third-order selection [112]. We calculated Manly selection ratios in which used values were proportions within a given habitat category of all GPS locations during the 7-day calving period (median 85 points per individual, range: 83–86 points) and available values were proportional areas of the same habitat category within the annual home range. Given our study focus on calving site selection in relation to logging and fire disturbance, and their patchiness in time and space across our study area and within individual moose annual home ranges, we conducted a targeted analysis of calving site selection as a function of time since fire and time since forestry. The timing of forestry and fire disturbance was assessed using a 30 m Landsat-derived forest change dataset from Hermosilla, A., C., C., W. and Campbell [106] and as updated by Pelletier, Cardille, Wulder, White and Hermosilla [107], which identifies areas across Canada’s forested ecosystems that were logged or burned each year between 1985 and 2022. Disturbance timing was expressed relative to when each moose was collared, and categorized as <1 year, 1–9 years, or 10–15 years before collaring. Habitats classified by Hermosilla et al. [106] and Pelletier, Cardille, Wulder, White and Hermosilla [107] as disturbed >15 years prior to collar deployment were assigned as forest or non-forest habitat according to Hermosilla, Wulder, White, Coops and Hobart [108]’s land cover map (Table S2.2). This targeted analysis was limited to females with <15-year-old disturbance features present within their annual home ranges, including 46 females and 62 calving events with <15-year-old forestry disturbance and 40 females and 60 calving events with <15-year-old fire disturbance present within the annual home range. Restricting this particular analysis to individuals with <15-year-old disturbance present in their annual home ranges allows us to directly and explicitly assess whether different disturbance types and ages are selected, avoided or used in proportion to availability. This contrasts the broader analysis that includes all individuals and all habitat features present on the landscape, thereby including disturbance history implicitly as reflected in the current landcover but not explicitly as a time since disturbance variable. Similar to the prior Manly selection ratio analysis, used values were proportions of all GPS locations during the 7-day calving period within <1-year, 1–9 year, and 10–15-year old cuts or burns (or forest or non-forest with no mapped disturbance within the last 15 years), while available habitats were proportional areas from within the annual home range occupied by the same disturbance categories.

We modelled calving site selection in relation to landcover, topography and road density, using a binomial logistic regression generalized linear model (GLM). Logging and fire disturbance were not included as predictors in this analysis due to their patchiness in time and space across our study area and within individual moose annual home ranges. The GLM was based on the same 7-day calving period used locations but with availability calculated from random points generated within each individual annual home range (n = 850 per individual). The response variable was coded as 1 for used locations and 0 for available locations. The collinearity of each habitat variable was tested via an adjusted Generalized Variance Inflation Factor (GVIF), accounting for differences in degrees of freedom across the categorical variables and calculated as GVIF1/(2df), where df is the degrees of freedom [113] and a GVIF1/2df of 2.24 or more generally shows multicollinearity [114]. Models gradually increasing in complexity were tested using Aikaike Information Criterion (AIC) and Nagelkerke’s pseudo R-squared values using the AICcmodavg [115] and pscl [116] R packages. Marginal predictions of the probability of use of the different habitat categories were calculated based on the best-supported model using the marginaleffects [117] R package. Individual variation in calving site characteristics was explored using a principal component analysis (PCA). This multivariate analysis focused on proportional use, during the 7-day calving period, of landcover and topography categories. Road density and disturbance was excluded from the PCA due to small effect sizes in GLM analyses, the absence of medium and high road densities in the northern study area, and the rarity of female use of fire- and forestry-disturbed habitats. Used proportions were standardized to ensure comparability among variables [118] and Eigen analysis of the covariance matrix was used to extract principal axes and the proportion of variance explained by each axis [119]. Because the PCA was an exploratory analysis and not a test of statistical significance, we included the habitat used by 53 females over 76 calving seasons in thisanalysis.

## 3. Results

**Figure 2:**
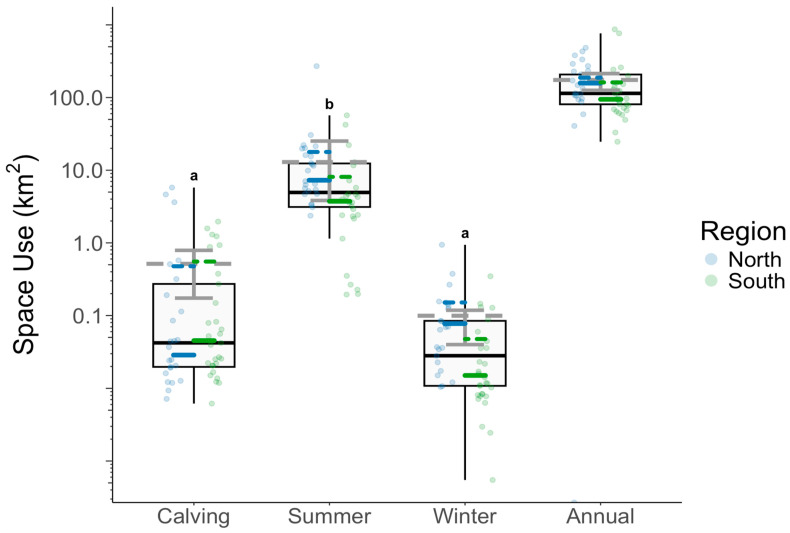
Calving space use (km2) measured using 95% minimum convex polygons for 53 7-day calving events of 53 female moose collared in the north (29 females) and in the south (24 females) of the study area, compared to 7-day winter, 7-day summer, and annual space use (logarithmic y-axis scale). Medians are shown as solid horizontal black lines, means as dotted horizontal grey lines accompanied by 95% confidence intervals, 50% interquartile ranges as boxes, and 1.5×inter-quantile range as vertical lines. Regional medians are shown as solid blue (north) or green (south) lines and means as dotted blue (north) or green (south) lines Unique letters above boxes indicate statistically significant differences (*p* < 0.05) between seasonal 7-day space use, based on post hoc Tukey’s HSD tests, with annual space use excluded from the statistical comparison due to a much longer sampling period.

Estimated parturition dates for 53 females (24 south and 29 north) over 76 calving seasons (38 south and 38 north) ranged from 18 May to 31 May. Median space use during the 7-day calving period was 0.04 km^2^ (mean 0.48 km^2^, range <0.01–5.77 km^2^; n = 53 females), which was similar to 7-day winter space use (median 0.03 km^2^, mean 0.08 km^2^, range <0.01–0.94 km^2^, n = 53 females), but smaller than 7-day summer space use (median 4.95 km^2^, mean 14.51 km^2^, range 0.19–270.41 km^2^, n = 47 females; or, with a single outlier female with 270.41 km^2^ space use removed, median 4.73 km^2^, mean 9.28 km^2^, range 0.19–56.88 km^2^) and much smaller than annual space use (median 113.87 km^2^, mean 169.88 km^2^, range: 24.63–866.56 km^2^, n = x females; Figure 2). Eight of the 53 space use estimates during the calving period exceeded 1 km^2^, with seven of these larger ranges driven by movement occurring on or around the estimated day of parturition, and one individual characterized by a ~4 km displacement 3 days after parturition followed by a return to its original area within 5 days after parturition. Seasonal differences in space use were significant (F_2,153_ = 7.24, *p* < 0.001 with all females included; F_2,150_ = 34.24, *p* < 0.001 with single outlier with a 270 km^2^ MCP removed, bringing max space use < 57 km^2^), with summer significantly larger than winter and calving, but calving and winter not significantly different from each other. Calving space use did not differ significantly between the north (median 0.04 km^2^, mean 0.63 km^2^, range <0.01–5.77 km^2^) and south (median 0.03 km^2^, mean 0.27 km^2^, range <0.01–1.58 km^2^; t_27.6_ = 1.04, *p* = 0.31). Estimation of space use using alternate 50% and 95% kernel density methods resulted in similar absolute estimates of seasonal space use and the same relative seasonal differences (Supplementary Materials S1).

### 3.1. Space Use and Fidelity

**Figure 3:**
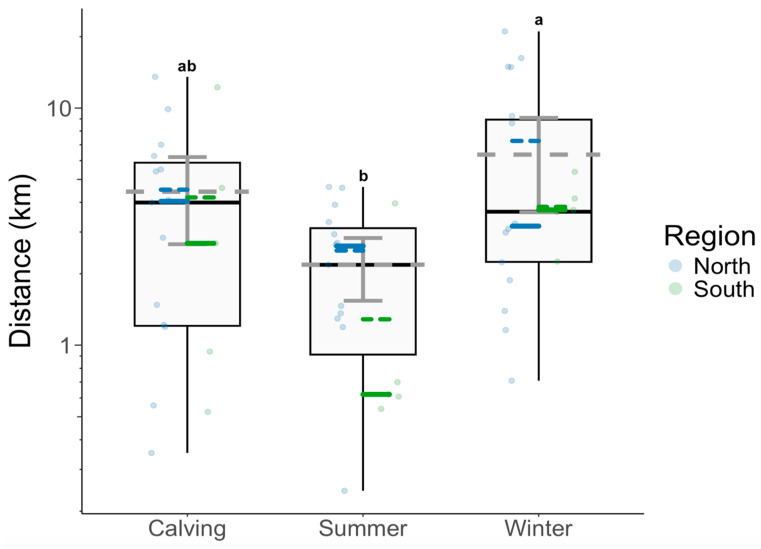
Calving site fidelity of 19 female moose collared in the north (14 females) or the south (5 females), measured as Euclidean distance (km) between the geometric centroids of locations during 7-day calving periods in two consecutive years, compared to 7-day summer and winter range fidelity (logarithmic y-axis scale). Medians are shown as solid horizontal black lines, means as dotted horizontal grey lines accompanied by 95% confidence intervals, 50% interquartile ranges as boxes, and 1.5×inter-quantile range as vertical lines. Regional medians are shown as solid blue (north) or green (south) lines and means as dotted blue (north) or green (south) lines. Unique letters above boxes indicate statistically significant differences (*p* < 0.05) between seasonal fidelity, based on post-hoc Tukey’s HSD tests.

Median calving site fidelity was 4.00 km (mean 4.44 km, range 0.35–13.54 km), showing lower fidelity than that between summer ranges (median 2.18 km, mean 2.18 km, range 0.24–4.65 km) but similar to fidelity between winter ranges (median 3.65 km, mean 6.36 km, range 0.70–21.05 km; Figure 3). Seasonal differences in fidelity were significant (F_2,62_ = 5.72, *p* = 0.005), with winter significantly different from summer, but no significant differences between calving and winter and between calving and summer. Calving site fidelity did not differ significantly between north (median 4.04 km, mean 4.53 km, range <0.35–13.5 km) and south (median 2.69 km, mean 4.20 km, range <0.52–3.96 km); however, these comparisons are informed by a relatively small sample size (19 females total, including 14 females in the north but only 5 females in the south). The median distance between the winter range and the calving site was 5.06 km (mean 5.91 km, range 0.31–15.30 km), that between the calving site and the summer range was 4.82 km (mean 12.83 km, range 0.34–343.99 km; or, with a single outlier with 344 km movement removed, median 4.60 km, mean 5.93 km, 0.34–18.54 km), and that between the winter range and the summer range was 5.10 km (mean 13.06 km, range 0.85–330.22 km; or, with a single outlier with 330 km distance removed, median 5.06 km, mean 6.45 km, 0.85–26.78 km). These between-season, within-year distances did not differ significantly from each other (F_2,147_ = 0.52, *p* = 0.593 with all females included; F_2,144_ = 0.31, *p* = 0.731 with a single outlier with a 344 km distance removed), and were similar in the northern and southern study areas for calving-to-summer and summer-to-winter distances but winter-to-calving distances were significantly longer in the north (median 7.16 km, mean 7.78 km, range 1.93–15.30 km) than in the south (median 3.81 km, mean 4.24 km, range 0.31–11.85 km; t_40.2_ = 3.64, *p* < 0.001).

**Figure 4:**
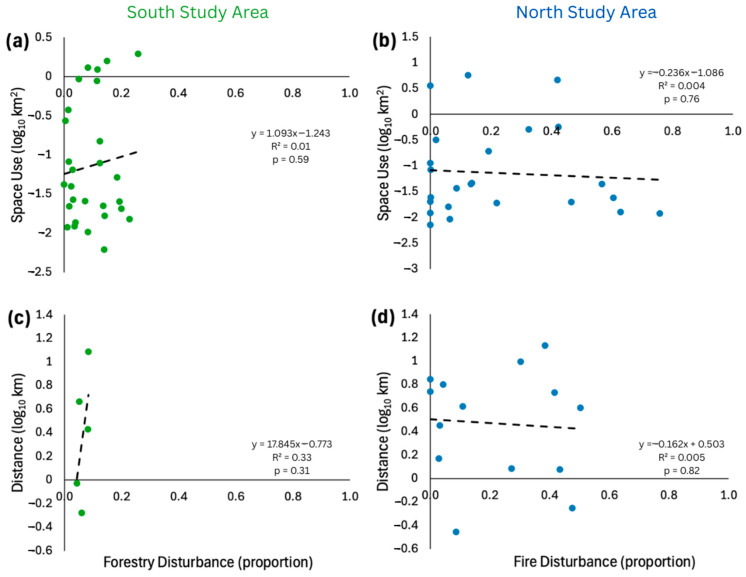
Scatter plots and linear regressions relating calving space use (**a**,**b**) or calving site fidelity (**c**,**d**) in relation to forestry disturbance in the south study area ((**a**,**c**), green symbols) or fire disturbance in the north study area ((**b**,**d**), blue symbols) within a 6 km radius of the calving site. Dashed lines indicate non-significant relationships.

Calving space use and calving site fidelity were not related to the extent of forestry disturbance around calving sites in the south or the extent of fire disturbance around calving sites in the north. The explanatory power and significance of linear regressions relating space use to forestry disturbance in the south (R^2^ = 0.01, *p* = 0.59) and fire disturbance in the north (R^2^ = 0.004, *p* = 0.76) and fidelity to forestry disturbance in the south (R^2^ = 0.33, *p* = 0.31) and fire disturbance in the north (R^2^ = 0.005, *p* = 0.82; Figure 4), were all low and non-significant.

### 3.2. Calving Site Selection

**Figure 5:**
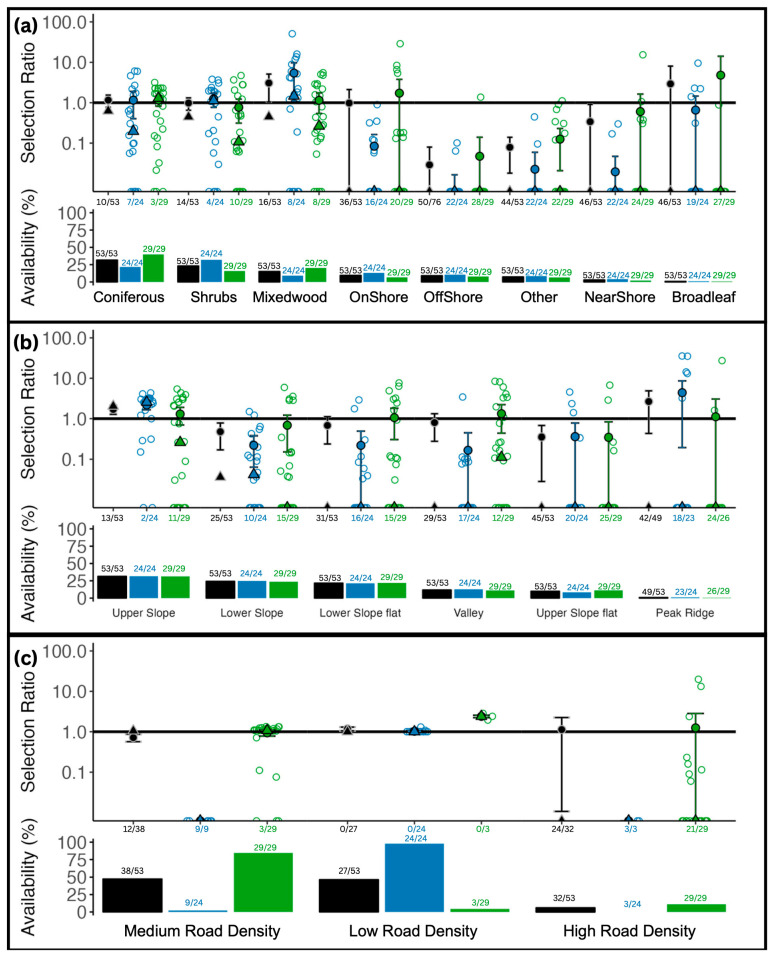
Manly selection ratios during calving (**top**) and annual home range availability (**bottom**) for (**a**) land cover, (**b**) topography, and (**c**) road density categories, displayed for female moose across the study area (black), in the north (blue) and in the south (green). Selection ratio plots include individual ratios (open circles), means (closed circle) accompanied by 95% confidence intervals, and medians (triangles), with the horizontal line separating selection (>1) from avoidance (<1); numbers along the x-axis indicate the proportion of females with no usage of a given category despite it being present in their home range, resulting in a selection ratio = 0. Availability bar charts represent the % area occupied by a given category averaged across the annual home ranges of all individuals, ordered by availability for the entire study area (black bars); numbers at the top of each bar indicate the proportion of females for which the category was present within their annual home range.

Calving site selection analyses, comparing habitat use during the 7 days post-calving relative to annual home range habitat availability, indicated use exceeded availability (mean selection ratio >1) for elevated areas, including upper slopes (mean selection ratio 1.7), which were common in annual home ranges (31%), and peak ridges (2.7), which were rare (1%; Figure 5a). Mixed-wood forests (3.1), which were intermediately available in the landscape (15%), and broadleaf forests (2.9), which were rare (1%), were also used, on average, more than they were available (Figure 5b). Most females avoided calving sites on lower slopes (selection ratio 0.7; availability 24%) and upper slope flats (0.4; 10%) and in nearshore (0.3; 3%) and offshore (<0.1; 9%) areas. Females in the south exhibited stronger selection of valleys (1.3; 11%) and onshore habitats (1.4; 7%) than females in the north (valleys 0.2; 12%, onshore 0.1; 13%). However, individual variation was high, with 95% confidence intervals overlapping 1.0 (indicating use proportional to availability) for all features, except for mixed-wood and upper slope forests, which were positively selected, and lower slope, upper slope flat, nearshore, other and offshore areas, which were avoided. In the south, females selected calving sites in low road density areas (2.4; 4%) and used medium (0.9; 85%) and high road density (1.2; 11%) areas proportionately to availability (Figure 5c). In the northern region of the study area, low road density was used proportionately to availability (1.0; 98%), medium road density was scarce (0; 2%), and high road density was absent (<1%).

**Figure 6:**
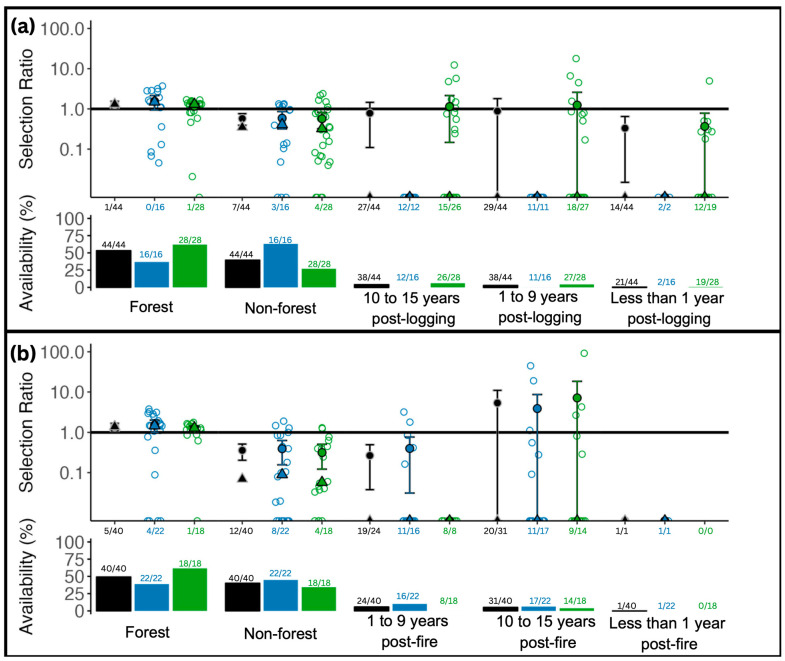
Manly selection ratios during calving (**top**) and annual home range availability (**bottom**) in habitats that are regenerating after (**a**) forestry disturbance and (**b**) fire disturbance displayed for female moose across the study area (black), in the north (blue) and in the south (green). Selection ratio plots include individual ratios (open circles), means (closed circle) accompanied by 95% confidence intervals, and medians (triangles), with the horizontal line separating selection (>1) from avoidance (<1); numbers along the x-axis indicate the proportion of females with no usage of a given category despite it being present in their home range, resulting in a selection ratio = 0. Availability bar charts represent the % area occupied by a given category averaged across the annual home ranges of all individuals, ordered by availability for the entire study area (black bars); numbers at the top of each bar indicate the proportion of females for which the category was present within their annual home range.

Calving site selection analyses focused on fire and forestry disturbance indicated females in the south avoided <1 year old cuts (0.4, 1%) and used 1–9-year-old cuts (1.2, 4%) and 10–15-year-old cuts (1.1, 6%) in proportion to their availability (Figure 6a). Females in the south and north avoided < 1 year old burns (0; <1%) and 1–9-year-old burns (0.3, 6%) but used 10–15-year-old burns (5.3, 5%) more than their availability, although confidence intervals overlapped with use proportional to availability. There were no notable differences in use of burn areas between the study areas (Figure 6b).

The best supported GLM of calving site selection included landcover, topography and road density, with landcover generally the strongest predictor of selection and road density the weakest (Supplementary Material Table S3.1). Marginal predictions of the probability of use based on the best supported model indicated high probability of use of mixed-wood and broadleaf landcover, peak ridge and upper slope topography, and medium and low road density (Figure S3.1). Marginal predictions indicated moderate probability of use for coniferous, shrubs, and onshore landcover, as well as lower slope flat, upper slope, and valley topography. Finally, marginal predictions indicated low probability of use of nearshore and offshore landcover, lower slope and upper slope flat topography, and high road density.

**Figure 7:**
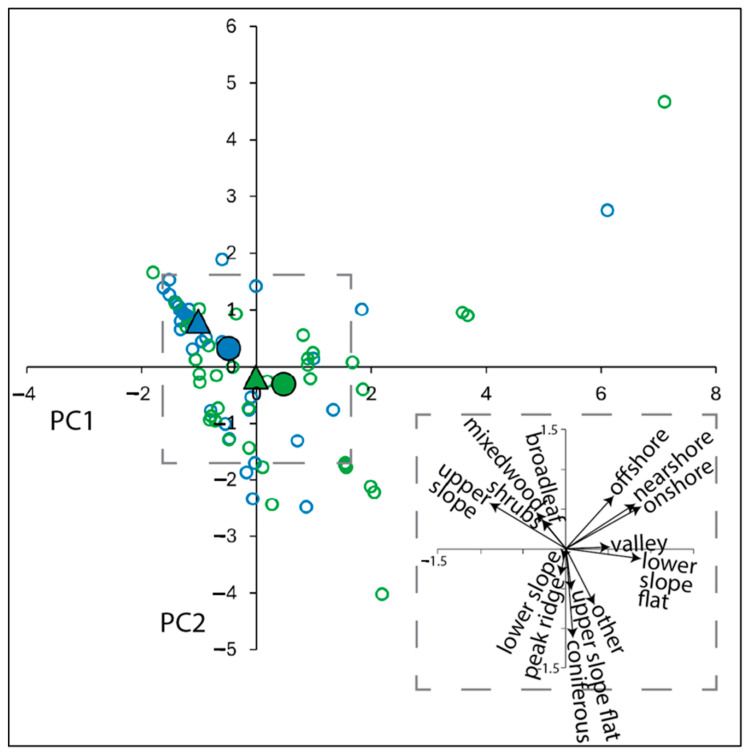
Principal component analysis (PCA) biplot showing proportional habitat use during the 7-day calving period by females in the south (green) and north (blue), including individual moose (open circles), means (closed circle), and medians (triangles). The bottom right inset displays landcover and topography category loadings in relation to PC1 and PC2.

Principal component analysis of individual variation in calving site characteristics related to land cover, topography, and waterbodies identified a PC1 driven primarily by topography, which accounted for 19.4% of total variance, and a PC2 driven primarily by land cover, which accounted for 13.4% of total variance (Figure 7). Although these two principal components collectively accounted for a relatively low proportion of total variance (32.8%; PC3 accounted for an additional 11.2%, resulting in a cumulative 44% variance accounted for by PC1-3), calving site use by most females was positioned towards upper slopes, mixed-wood forest, and broadleaf forest. However, calving site use by some females was positioned away from these habitats and towards either peak ridges, upper slope flats, and coniferous stands or nearshore and onshore valleys and lower slope flats. Calving sites used by females in the north were concentrated towards mixed-wood, broadleaf, shrubs and upper slopes, while those in the south were more dispersed across these habitat types, as well as shore-associated lowlands, and upland associated coniferous forests (Figure 7).

## 4. Discussion

Moose space use around calving sites for the 7 days following parturition was highly spatially restricted, with a median area of 0.04 km^2^, equivalent to the area of a 225 m radius circle, which was similar to the 0.02 km^2^ reported by McGraw, Terry and Moen [41] and other sources describing restricted space use [44,45,57]. Though we found 7-day calving space use to be highly spatially restricted, space use during winter, measured for an equal-duration 7-day period and with a similar number of locations, was almost as small and not significantly different from calving space use, and both were significantly smaller than summer space use. Moose had a median annual home range of 113.87 km^2^, equivalent to the area of a 6.02 km radius circle. Calving site fidelity among females observed over two calving seasons was significantly less than summer site fidelity and similar to winter site fidelity expressed by the same females and estimated in a similar manner. The 4 km calving site fidelity we document here is similar to the 3.15 km calving site fidelity reported by Welch, Rodgers and McKinley [59]. Previous studies have reported high fidelity of moose locations throughout the year [60,120,121]. In our study, moose moved a median straight-line distance of 5.06 km between late winter locations (21 March to 27 March) and their calving site, 4.82 km between calving site and summer locations (1 August to 7 August), and 5.10 km between late-winter and summer locations. Some moose were characterized by much longer distances travelled between winter and summer ranges, including one individual characterized by >300 km distance traveled. While this range of distances traveled between winter and summer ranges could result from some but not all female moose undergoing seasonal migrations [122,123,124], in our study population, large-scale movements are uncommon, occur at variable times of year, and are rarely followed by seasonally consistent return travel (Brodeur, unpublished data). Calving site space use and fidelity was similar between the southern study area affected by forestry disturbance and the northern study area affected by fire disturbance. However, the distance between winter range and calving sites was longer in the northern study area than the southern study area, which could reflect the lower abundance and therefore longer distances between high-habitat-quality patches in the north. Calving site space use and fidelity was unrelated to the extent of forestry and fire disturbance in annual home range areas around calving sites.

Calving site selection analyses, comparing habitats used during the 7 days after calving relative to habitats available in annual home ranges, indicated positive selection of mixed-wood forests and avoidance of offshore areas and other habitats. Females generally avoided calving in areas disturbed by fire or forestry within the last year but calved in 10- to 15-year-old burns more than they were available. Further evidence of the tendency for females to avoid calving in areas surrounded by extensive forestry disturbance and their tolerance for calving in areas surrounded by extensive fire disturbance is indicated by the differing ranges of proportional forestry (range 0–0.26) and fire (range 0–0.76) disturbance present within a 6 km radius of the calving site. There was extensive individual variation in patterns of selection, as highlighted by selection ratios that generally overlapped with 1 (indicating use in proportion to availability) and our principal component analysis showing that although most females selected calving sites in mixed-wood or broadleaf stands located on upper slopes, some females calved in lowlands and near water, including in coniferous stands located in flatter areas or peak ridges. That the first principal component was dominated by topography, even though calving site selection was most influenced by land cover, suggests that topography better accounts for individual variability in selection, whereas landcover better accounts for among-individual consistency in selection. Similarly, Poole, Serrouya and Stuart-Smith [44] described two different strategies expressed by female moose at calving, where females either chose elevated areas further from water or low-lying areas close to water. Local Cree Knowledge also highlights the use of swampy and riparian zones for calving [90,92,93] and Jacqmain, Dussault, Courtois and Bélanger [92] reported collared moose preference for calving in alder stands. However, our GLM marginal predictions do offer evidence of an overall tendency for calving site selection on peak ridges in mixed-wood and broadleaf forests. Although recent cut blocks and burned areas (less than 1 year old, 1- to 9-year-old cuts) were avoided during calving, females, on average, calved in 10- to 15-year-old burns more than they were available. However, individual variability was high, indicated by a 95% confidence interval overlapping with use proportional to availability. Previous studies report that moose avoid calving in 1- to 8-year-old cutblocks [62] and 0-to10-year-old salvaged areas [87]. Calving in 10- to 15-year-old burns could be associated with the abundance of cover and forage present in the sub-canopy of regenerating burns [75,77].

Although regenerating cuts are also often associated with abundant cover and forage, females did not express the same tendency for calving in 10- to 15-year-old cut blocks as they did in 10- to 15-year-old burns. Our analysis did not consider stand composition prior to disturbance; disturbed stands that were originally mixed-wood and broadleaf, which are less common in the study area, may recover more quickly than disturbed stands that were originally coniferous, which are more common in the study area. Thomas, Reid, Barclay and Jung [87] reported an elevated probability of females calving in 11–25-year-old salvaged areas. Mumma, Gillingham, Marshall, Procter, Bevington and Scheideman [62] suggested regenerating cuts may be less attractive to moose because of a lack of variety in regrowing vegetation composition. Cree land-users knowledgeable about moose habitat selection in our study area recognize the use of regenerating cuts by moose but consistently suggest regenerating burns are higher-quality habitat for moose than regenerating cuts, due to the silviculture and scarification treatments, as well as the high road densities associated with forestry practices in the region [90]. We found that within the commercial forestry zone, where road density varied from low to high, moose preferentially calved in areas of low-road-density, whereas medium- and high-road-density areas were used in proportion to their availability. Calving in areas of low-road-density has been attributed to avoiding roads, facilitation of predator movement and high levels of human disturbance [86]. We did not find evidence that calving site space use and fidelity varied according to the extent of forestry or fire disturbance around calving sites. In contrast, Welch, Rodgers and McKinley [59] found higher levels of disturbance were associated with lower calving site fidelity.

Many females calved in elevated areas, including peak ridges and upper slopes, away from lower-lying areas including lower slopes. Many studies emphasize protection from predators as a primary attribute of moose calving sites [37,41,42,44,53] and a few studies have suggested predators may be less likely to encounter calving sites, or may be detected earlier, when approaching calving sites located in elevated areas. However, the only study we are aware of that has directly assessed how elevation affects moose predation is Kunkel and Pletscher’s [125] winter-focused field work showing that wolf kill sites occur at lower elevations than expected based on moose collar locations. Upland calving sites were more consistently used in the northern study area than in the southern area, which may reflect mixed-wood and broadleaf stands, and the combination of cover and forage that they offer [52], which are more restricted to upland areas in the northerly zone. Most calving sites were not associated with permanent water bodies, possibly because of the increased detectability of cows and calves in proximity to these open areas [37]. Females and their calves use more shallow aquatic habitats later in the summer [52,91], when calves are older and better able to walk and swim in water [20,50] and when aquatic habitats offer more forage and relief from insect harassment [52,126].

## 5. Conclusions

Our findings highlight that mixed-wood and broadleaf stands occurring on peak ridges and upper slopes are used and selected by female moose as calving sites. Accordingly, mixed-wood and broadleaf uplands, which are relatively uncommon landscape features in this region, provide preferred habitat for moose during late winter [90,91] as well as during calving, with both periods recognized as critical phases in the annual cycle, when moose are highly localized, vulnerable to predators, and have specialized habitat requirements [37,41,42,52,53,91,127,128,129]. Our results also show that areas disturbed by fire or forestry can become preferentially used calving sites 10 years after fire but not within 15 years after forestry. Furthermore, our results show females preferentially calve in low-road-density areas, which occur away from forestry activities, where linear features persist in the landscape long after forestry activities are completed [130]. We found calving site fidelity was relatively low, with straight-line distance between subsequent year calving sites ranging from less than 1 km to more than 13 km, which precludes an individual-based calving site approach to habitat protection. Similarly, although the tendency for calving to occur in mixed-wood and deciduous stands mirrors the habitat preferences expressed by females in winter and summer (see Stern [91]), given the multi-kilometre seasonal relocations quantified here, protecting the specific stands used by females in one season (e.g., winter) is unlikely to protect the stands they will use in subsequent seasons (e.g., calving). Instead, habitat and population management should focus on maintaining the types of habitats that moose preferentially use during critical annual periods, which we show here to include elevated mixed-wood and broadleaf stands as preferred calving sites but also to extend to a wider diversity of calving habitats, including lowlands near water and regenerating burns.

## Data Availability

Maps for landcover categories and time since disturbance were accessed via “https://opendata.nfis.org/mapserver/nfis-change_eng.html” (accessed in 2 February 2025). Maps for topography were accessed via the following link: “https://developers.google.com/earth-engine/datasets/catalog/CSP_ERGo_1_0_Global_SRTM_landforms#description” (accessed on 2 February 2025). The polyline road map named “Routard” was accessed via internal Government of Quebec database, but can also be accessed under the name “AQReseau” via “https://www.donneesquebec.ca/recherche/dataset/adresses-quebec” (accessed on 2 February 2025). Moose location data is considered sensitive because this species is subject to harvest. Locations of harvested species of potential conservation concern cannot be shared in the public domain.

## Supplementary Materials

The following supporting information can be downloaded at: https://www.mdpi.com/article/10.3390/ani16111614/s1, Section S1: Supplementary Analysis of Space Use; Figure S1.1: Boxplots showing median (lines), 25th and 75th percentiles (boxes), 10th and 95th percentiles (whiskers), and outliers (points) of female space use estimated using three methods (95% MCPs, 50% kernel density, 95% kernel density) across three seasons (calving, summer, winter). With GPS collars programmed to record locations at a 2-h fix rate and a 7-day period, each individual estimate is informed by approximately 84 locations; Figure S1.2: Scatter plots comparing 95% MCPs and 95% kernel density estimates of 7-day space use of A) 53 female moose during the calving season, B) 47 female moose in summer, and C) 53 female moose in late winter; Figure S1.3: Histogram of 7-day moose space use, estimated using 95% MCP’s, dividing females into six space use bins (smallest on left, largest on right), with a map of a randomly selected female from each bin, showing all GPS locations (black dots), 95% kernel density polygon (pink), 50% kernel density polygon (red), and 95% MCP’s (blue) for that randomly selected female. Scale bar varies between maps as space use increases from the lowest to the highest bin. A) 53 female moose during the calving season, B) 47 female moose in summer, and C) 53 female moose in late summer; Section S2: Habitat variable reclassification and adjustment procedures Table S2.1: Summary of habitat variable reclassification and adjustment procedures for landcover, topography and road density layers used in moose habitat selection analyses; Table S2.2: Summary of habitat variable reclassification and adjustment procedures for disturbance categories including time since logging and time since fire; Section S3: Additional results; Table S3.1: All combinations of logistic regression binomial generalized linear models (GLMs) (used vs available) for the selection of three habitat variables during calving (i.e., topography, landcover and road density) ordered by descending model fit on ΔAIC and Nagelkerke Pseudo R2. Figure S3.1: Predicted probability of use for Landcover (A), Topography (B) and Road density (C) based on best GLM model with horizontal line at 0.5 separating high chances of use and low chances of use.
