# Supplementary material for: Moose Space Use, Fidelity, and Selection of Calving Sites Within Forestry- and Fire-Disturbance Regimes of Northern Quebec, Canada
Source: Animals (Basel). 2026 May 26;16(11):1614. doi: 10.3390/ani16111614 (PMC13255596; doi:10.3390/ani16111614)
Supplement: Supplementary file 1 [file animals-16-01614-s001.zip › animals-4267046-supplementary.pdf]

Supplementary Material

S1. Supplementary Analysis of Space Use

In the primary analysis presented in the main text, we used 95% minimum convex polygons (MCPs), estimated with the QGIS animove plugin, to quantify and compare space use during a 7-day late winter period (from March 21 to March 27), a 7-day calving period (including the parturition date and the next 6 days thereafter), and a 7-day summer period (from August 1 to August 7). Here we also quantify space use using a 50% and 95% kernel density measure, estimated using the adehabitatHR package in R, and compare results obtained from the three space use estimators (95% MCPs, 50% kernel density, 95% kernel density) across the three seasons.

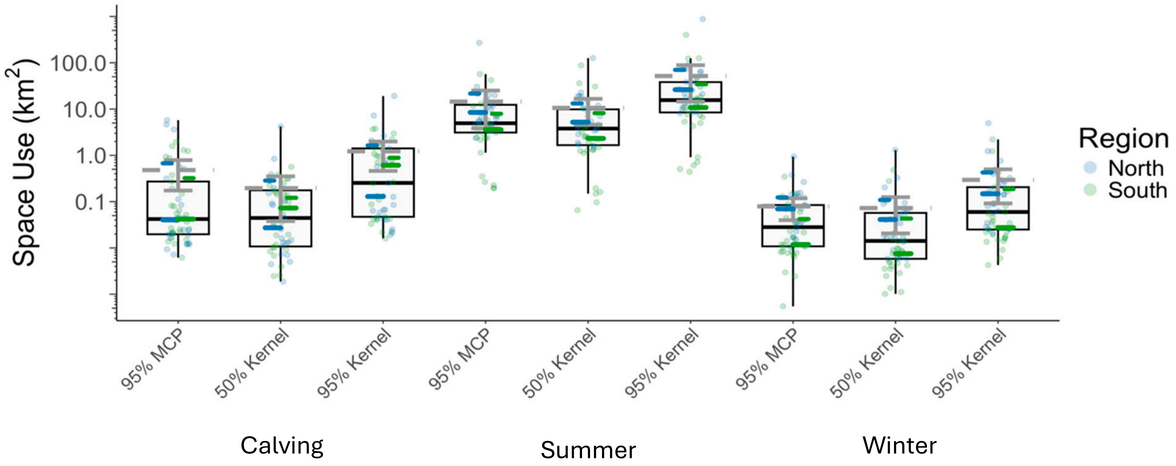

Figure S1.1. Boxplots showing median (lines), 25th and 75th percentiles (boxes), 10th and 95th percentiles (whiskers), and outliers (points) of female space use estimated using three methods (95% MCPs, 50% kernel density, 95% kernel density) across three seasons (calving, summer, winter). With GPS collars programmed to record locations at a 2-hour fix rate and a 7-day period, each individual estimate is informed by approximately 84 locations.

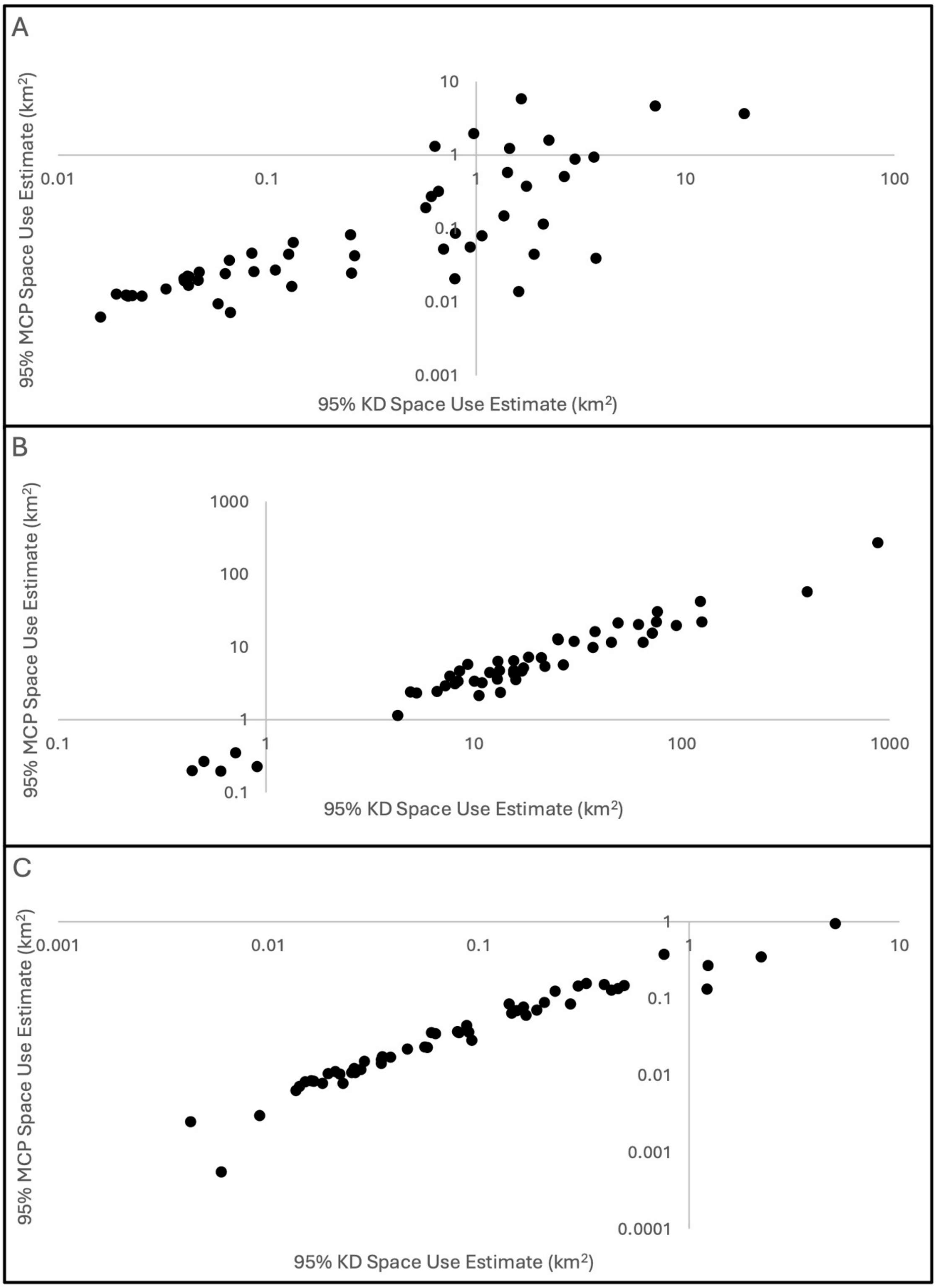

Figure S1.2. Scatter plots comparing 95% MCPs and 95% kernel density estimates of 7-day space use of A) 53 female moose during the calving season, B) 47 female moose in summer, and C) 53 female moose in late winter.

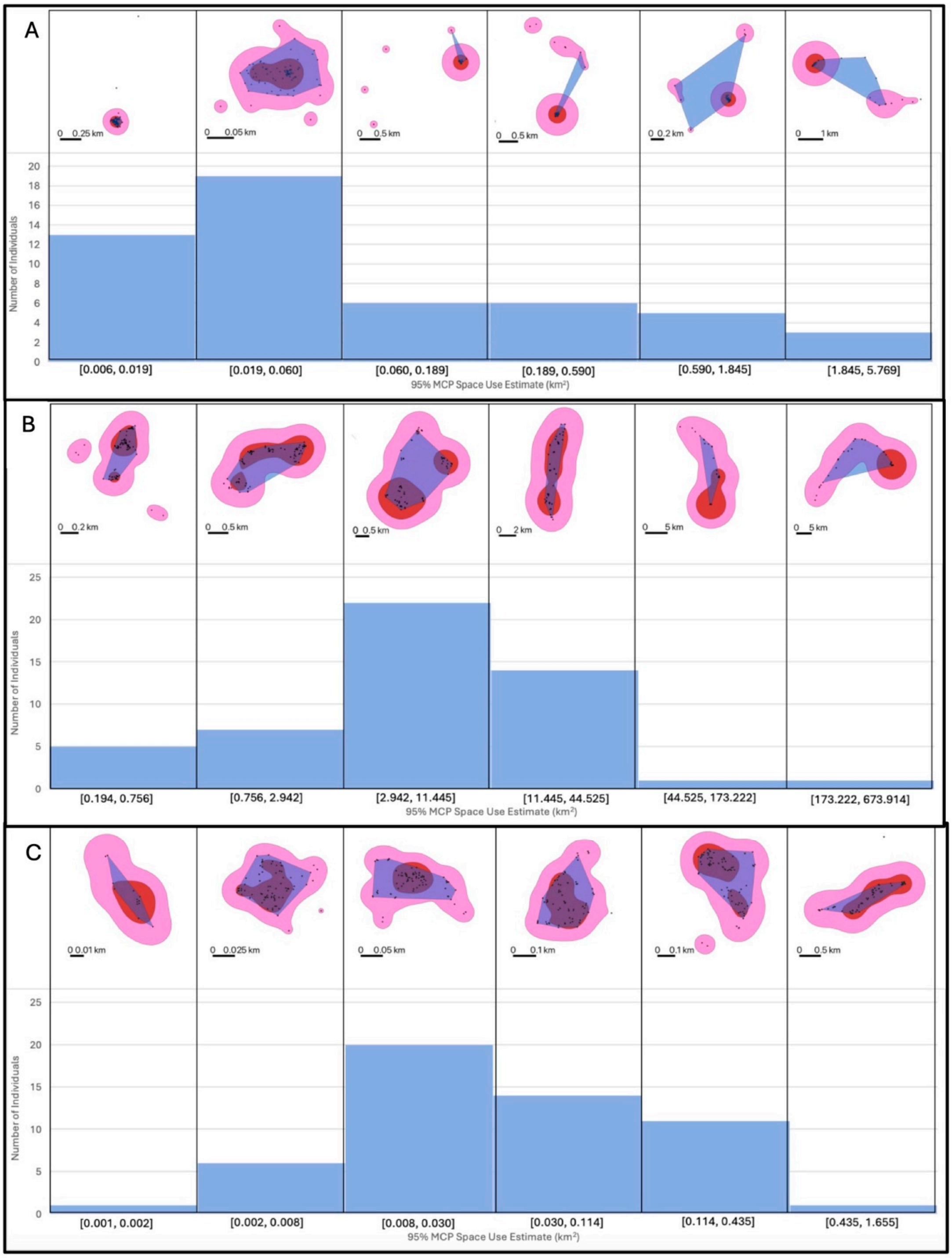

Figure S1.3. Histogram of 7-day moose space use, estimated using 95% MCP's, dividing females into six space use bins (smallest on left, largest on right), with a map of a randomly selected female from each bin, showing all GPS locations (black dots), 95% kernel density polygon (pink), 50% kernel density polygon (red), and 95% MCP's (blue) for that randomly selected female. Scale bar varies between maps as space use increases from the lowest to the highest bin. A) 53 female moose during the calving season, B) 47 female moose in summer, and C) 53 female moose in late summer.

S2 Habitat variable reclassification and adjustment procedures

Table S2.1 Summary of habitat variable reclassification and adjustment procedures for landcover, topography and road density layers used in moose habitat selection analyses.

Variable	Original Variable Description	Original Class	Adjusted Class	Adjusted Class Description	Source
Landcover	High-resolution Landsat-derived annual land cover maps at 30m resolution from 1984 to 2022 that were used to characterize forested ecosystems across Canada. Maps were generated using Landsat composites, disturbance information, and environmental variables, with post-processing via a Hidden Markov Model to ensure consistent land cover transitions over time. Maps from 2018 to 2022 were clipped to the extent of Eeyou Istchee for this study.	Water	Nearshore	Buffer of 25m in water from shoreline of water class	Hermosilla, *et al.* [1]
			Offshore	Erase Nearshore from water class and classify remaining water class as Offshore	
			OnShore	Buffer of 80m on land from shoreline of water class	
		Coniferous	Coniferous		
		Broadleaf	Broadleaf		
		Mixedwood	Mixedwood		
		Shrubs	Shrubs		
		Herbs	Other	Based on low used locations and low availability, categories, the remaining categories were combine as one class.	
		Bryoids			
		Wetland			
		Wetland-treed			
		Exposed/ Barren Land			
		Rock/ Rubble			
Topography	Landform classes (30m resolution) derived from 30m STRM DEM created by combining Continuous Heat-Insolation Load Index (SRTM CHILI) and the	Mountain/ divide		Not present in study area	Theobald, *et al.* [2]
		Cliff			
		Peak Ridge (warm)	Peak Ridge	Combine classes	
		Peak Ridge			
		Peak Ridge (cool)			
		Upper Slope (warm)	Upper Slope	Combine classes	

	multi-scale Topographic Position Index (SRTM mTPI) datasets. This multi-scale classification approach identifies ecologically meaningful landform types based on topographic position and solar exposure.The map was clipped to the extent of Eeyou Istchee for this study.	Upper Slope			
		Upper Slope (cool)			
		Upper slope flat	Upper Slope flat		
		Lower Slope (warm)	Lower Slope	Combine classes	
		Lower Slope			
		Lower Slope (cool)			
		Lower Slope flat	Lower Slope flat		
		Valley	Valley	Combine classes	
		Valley (narrow)			
Road Density	Polyline road network map obtained Quebec provincial Routard road layer available through intern Government of Quebec database. The map was clipped to the extent of Eeyou Istchee and included all roads regardless of their classification.	Roads	LowRoad	Calculated line density over a 1km radius and categorized in 3 bins using jenks normal distribution breaks (Low road 0 to 0.66 km/km2; Medium road 0.66 to 1.86 km/km2; HighRoad 1.86 to 15.34 km/km2)	Gouvernement du Québec [3]
			MediumRoad		
			HighRoad		

Table S2.2 Summary of habitat variable reclassification and adjustment procedures for disturbance categories including time since logging and time since fire.

Variable	Original Variable description	Original Classes	Adjusted Classes	Adjustments	Source
Time Since Logging	Landsat-derived forest change dataset, which identifies areas across Canada's forested ecosystems that were logged or burned each year between 1985 and 2020. The disturbances are detected through the analysis of shifts in spectral signatures caused by vegetation loss, with disturbance types differentiated based on the spatial patterns of change, all at a 30 m resolution. More recent maps for 2021 and 2022 were generated based on similar protocols. Maps from 2018 to 2022 were clipped to the extent of Eeyou Istchee for this study.	Logged areas categorized by year of harvest	Less than 1 year post-logging	Isolate and grouped areas logged less than a year from collaring	Hermosilla, *et al.* [4]; **Pelletier, *et al.* [5]**
			1 to 9 years post-logging	Isolate and group areas logged between 1 to 9 years before collaring	
			10 to 15 years post-logging	Isolate and group areaslogged between 10 to 15 years collaring	
			Forest	Combine logged areas older than 15 years before collaring and uncut land and assign, based on landcover variables in Table S1, forest (coniferous, broadleaf, mixedwood) or non-forest (Nearshore, Offshore, Shrubs, other)	
			Non-Forest		
Time Since Fire	As above	Wildfire areas categorized by year of occurrence	Less than 1 year post-fire	Isolate and group areas burned less than a year from collaring	Hermosilla, *et al.* [4]; **Pelletier, *et al.* [5]**
			1 to 9 years post-fire	Isolate and group areas burned between 1 to 9 years before collaring	
			10 to 15 years post-fire	Isolate and group areas burned between 10 to 15 years collaring	
			Forest		

			Non-Forest	Combine burned areas older than 15 years before collaring and uncut land and assign, based on landcover variables in Table S1, forest (coniferous, broadleaf, mixedwood) or non-forest (Nearshore, Offshore, Shrubs, other)	

S3 Additional Results

Table S3.1 All combinations of logistic regression binomial generalized linear models (GLMs) (used vs available) for the selection of three habitat variables during calving (i.e., topography, landcover and road density) ordered by descending model fit on ΔAIC and Nagelkerke Pseudo R2.

**Model**	**AIC**	**ΔAIC**	**Pseudo R2**
Used ~ Landcover +Topography + Road Density	5301.4	0	0.164
Used ~ Landcover + Topography	5311.5	10.1	0.162
Used ~ Landcover + Road Density	5530.3	228.9	0.124
Used ~ Landcover	5538.2	236.8	0.122
Used ~ Topography + Road Density	5863.5	562.1	0.067
Used ~ Topography	5888.5	587.1	0.062
Used ~ Road Density	6236.0	934.6	0.002

Marginal predictions of the probability of use based on the best supported model indicated high probability of use of mixedwood and broadleaf landcover, peak ridge and upper slope topography, and medium and low road density (Figure S3.1).

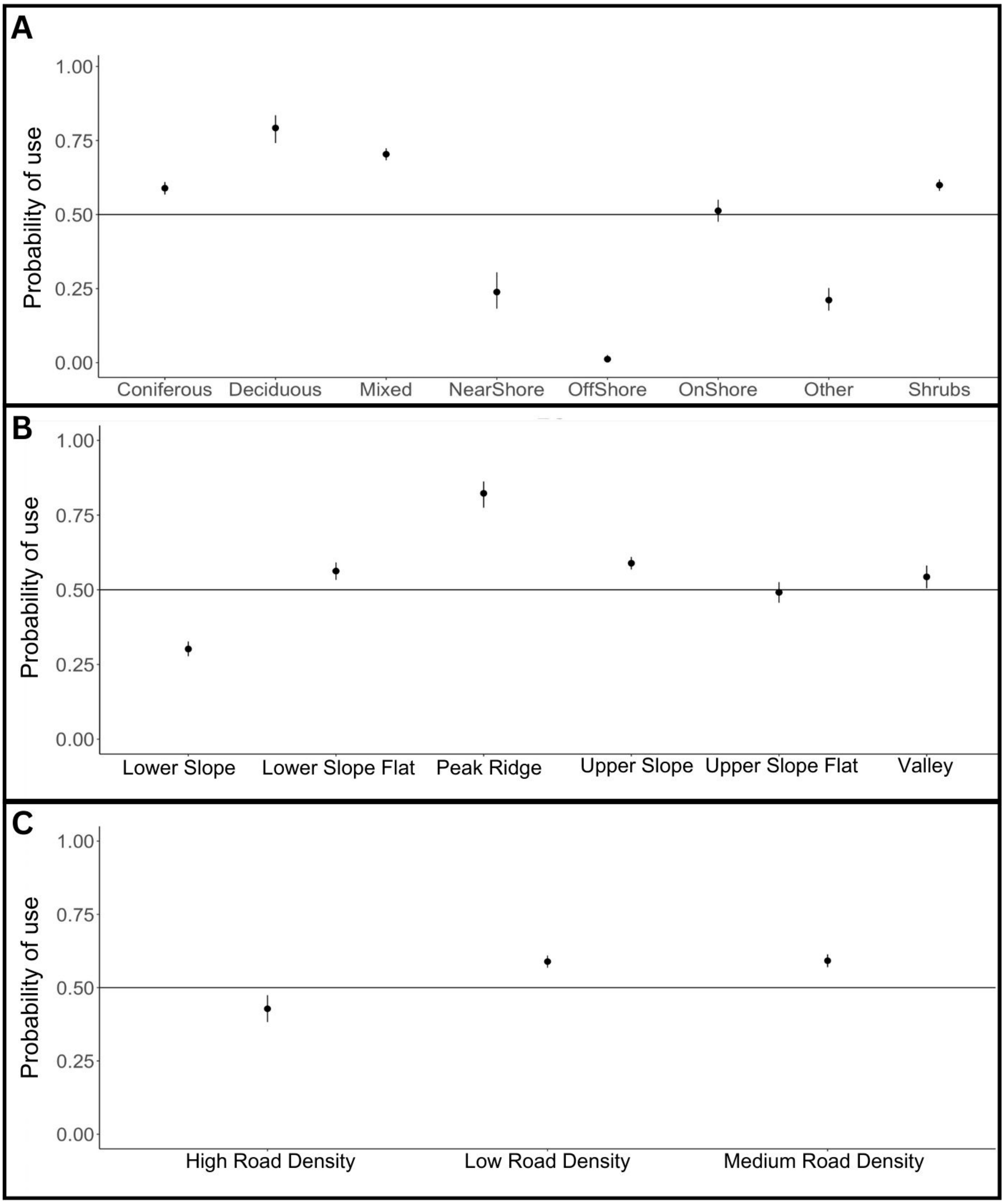

Figure S3.1. Predicted probability of use for Landcover (A), Topography (B) and Road density (C) based on best GLM model with horizontal line at 0.5 separating high chances of use and low chances of use.

References:

1. Hermosilla, T.; Wulder, M.A.; White, J.C.; Coops, N.C.; Hobart, G.W. Disturbance-Informed Annual Land Cover Classification Maps of Canada's Forested Ecosystems for a 29-Year Landsat Time Series. Canadian Journal of Remote Sensing 2018, 44, 67-87, doi:https://doi.org/10.1080/07038992.2018.1437719.
2. Theobald, D.M.; Harrison-Atlas, D.; Monahan, W.B.; Albano, C.M. Ecologically-Relevant Maps of Landforms and Physiographic Diversity for Climate Adaptation Planning. PLOS ONE 2015, 10, doi:https://doi.org/10.1371/journal.pone.0143619.
3. Gouvernement du Québec. Couche Routard. 2024.
4. Hermosilla, T.; A., W.M.; C., W.J.; C., C.N.; W., H.G.; and Campbell, L.B. Mass data processing of time series Landsat imagery: pixels to data products for forest monitoring. International Journal of Digital Earth 2016, 9, 1035-1054, doi:10.1080/17538947.2016.1187673.
5. Pelletier, F.; Cardille, J.A.; Wulder, M.A.; White, J.C.; Hermosilla, T. Inter- and intra-year forest change detection and monitoring of aboveground biomass dynamics using Sentinel-2 and Landsat. Remote Sensing of Environment 2024, 301, doi:https://doi.org/10.1016/j.rse.2023.113931.
